# Aristolochic Acid and Immunotherapy for Urothelial Carcinoma: Directions for unmet Needs

**DOI:** 10.3390/ijms20133162

**Published:** 2019-06-28

**Authors:** Huang-Yu Yang, Chih-Chao Yang, Chao-Yi Wu, Li-Jen Wang, Kun-Lin Lu

**Affiliations:** 1 Department of Nephrology, Chang Gung Memorial Hospital, College of Medicine, Chang Gung University, Taoyuan 333, Taiwan; 2 Division of Nephrology, Department of Internal Medicine, Kaohsiung Chang Gung Memorial Hospital and Chang Gung University College of Medicine, Kaohsiung 83301, Taiwan; 3 Division of Allergy, Asthma, and Rheumatology, Department of Pediatrics, Chang Gung Memorial Hospital, College of Medicine, Chang Gung University, Taoyuan 333, Taiwan; 4 Department of Medical Imaging and Radiological Science, College of Medicine, Chang Gung University, Taoyuan 333, Taiwan; 5 Department of Medical Imaging and Intervention, Chang Gung Memorial Hospital at Linkou, Taoyuan 333, Taiwan; 6 Department of Medical Education, Chang Gung Memorial Hospital at Linkou, Taoyuan 333, Taiwan

**Keywords:** upper tract urothelial carcinoma, urothelial carcinoma of the bladder, aristolochic acid, immune checkpoint inhibitors, mutation load

## Abstract

Urothelial carcinoma of the bladder (UCB) and upper tracts (UTUC) used to share management with similar principles. However, their genetic and epigenetic differences along with different responses to immunotherapy were recently identified, which are reminiscent of their distinct etiologies. Different from the variety of environmental factors relating to UCB, UTUC is best known for its close relationship with exposure to aristolochic acid (AA). AA is believed to cause its carcinogenicity through forming DNA adducts of deoxyadenosine-aristolactam, as well as A:T → T:A transversions in the *TP53* tumor suppressor gene. Since recent findings suggested that cancers with higher somatic mutations are associated with better treatment responses upon immune checkpoint blockade, UTUC and AA-related biomarkers reasonably serve as good candidates, as well as a potential prognostic predictor for the flourishing immunotherapy. This review covers the current state of the literature on the clinical response of UTUC and UCB receiving immunotherapy and points out directions for refinement regarding patient selection.

## 1. Introduction

Being the ninth most predominant malignancy worldwide [1], bladder cancer has an estimation of about 430,000 new cases diagnosed a year globally [2], with urothelial carcinoma of the bladder (UCB) being the most common histological type [3]. On the other hand, upper tract urothelial carcinoma (UTUC) is a malignancy affecting the lining of the urinary tract from the calyces to the distal ureter. Despite accounting for only 5% of all urothelial cancers [4], about 60% of UTUCs are invasive at diagnosis [5]. Although UTUC and UCB shared tobacco exposure as one of their main risk factors [6,7], the consumption of aristolochic acid (AA), which had been a common component of Chinese medicines and certain weight-losing medications, was identified as a depictive environmental factor associated more with UTUC rather than UCB [8]. In endemic areas, more than half of the UTUC cases were considered to be related to AA exposure based on molecular epidemiological evidence [9,10]. However, relatively little attention has been paid to address their prognosis in clinical scenarios.

## 2. The Link between AA and UTUC

AA is a natural alkaloid compound produced by the *Aristolochia* species of plants and was implemented as a part of Chinese medicines in Eastern countries, while it mainly served as a component of alternative medicine in Western countries for weight-loss or purely due to errors in plant collecting. However, AA has been known for its close relationship with Chinese herbal nephropathy in the East and Balkan endemic nephropathy in the West [9,11,12], both sharing the widespread interstitial sclerosis and tubular atrophy extending from the outer to the inner cortex as their pathological hallmarks [13,14,15]. Currently known as aristolochic acid nephropathy [16], this endemic disease may not only result in end-stage renal disease (ESRD), but also UTUC [8,17,18,19]. Therefore, it is no surprise that a consistent linkage between ESRD patients and their increased urothelial carcinoma (UC) risks has been repeatedly reported in AA-endemic area [20,21], with a general sequential order being UC after ESRD [22,23].

It has been well known that there is a significantly increased incidence rate for cancers among patients with ESRD in dialysis-dependent patients, especially for cancers of the kidney or the upper urinary tract, but not of the bladder, as compared with the general population [24,25,26]. However, when we look closer into the pathological type, UC popped up as the most common carcinoma related to patients suffering from chronic kidney disease or ESRD in AA-endemic areas [27,28,29], which is in clear contrast to other countries where renal cell carcinoma is the predominant one [30,31]. In Taiwan, it was estimated that one-third of their total population had ever consumed Chinese herbal products containing AA [32]. Therefore, many researchers have looked into this specific population, discovering that among 10,890 ESRD patients in Taiwan, chronic tubule-interstitial nephritis relating to long-term use of Chinese herbs accounted for 19.4% of this population, and the following incidence of UC was as high as 0.9%, with a recurrence rate of 35.7% [33]. The increased incidence of UC was still significantly related to a history of Chinese herb use after kidney transplantation in Taiwan [34,35]. In addition, a dose-dependent relationship was further demonstrated between the consumption of AA-containing Chinese herbal products and an increased risk of cancer of the urinary tract [36]. In clear contrast to non-endemic areas, UC in AA-endemic areas has consistently shown its slight female predominance with a more dramatic surge in the incidence of UTUC as compared to that of UCB [37,38,39,40]. In Taiwan, UTUC accounts for about 10–25% of all UC [41], and its recurrence has been linked to exposure history to AA, as well as impaired kidney function [42,43]. The distinct epidemiology, therefore, implies different underlying pathogenesis of a considerable part of UTUC that relates to a certain carcinogenicity of AA, especially within AA-endemic areas.

**Figure 1:**
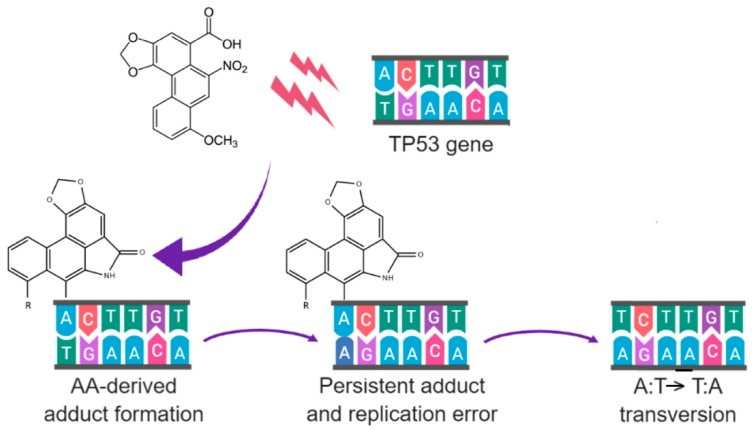
An example of AA-derived DNA adducts leading to gene mutations. After reductive metabolism, AA-derived DNA adducts are bound to the exocyclic amino groups of purine bases through their electrophilic cyclic *N*-acylnitrenium ion. If the adducts persisted through DNA replications, mutations, such as an A:T → T:A transversion in *TP53* gene, may occur.

As demonstrated by Grollman et al. [44], AA-derived DNA adducts were exclusively identified in all patients with Balkan endemic nephropathy. Within patients diagnosed with UTUC, only those from AA-endemic regions were found with AA-derived DNA adducts, accompanied by high frequencies of mutations of A:T pairs of the *TP53* gene. Further epidemiological research expanded by Jelaković et al. showed that AA-derived DNA adducts were present in 70.1% of UTUC patients living in AA-endemic areas [9], with a female-predominant trend. These DNA adducts persisted and can even be detected decades after exposure [45]. In addition, mutations in the *TP53* gene were identified in 40% of patients from AA-endemic areas, with over half of these mutations being an A:T → T:A transversion mutation [46]. Strikingly, 94% of these patients with A:T → T:A transversion mutations had concurrent AA-derived DNA adducts, reflecting the well-known intimate association between DNA adducts and gene mutations [47] (Figure 1). In the case of AA, these DNA adducts are believed to exert their carcinogenic effect through downregulation of DNA repair genes [48]. Similar findings in the Taiwan population echo the carcinogenic potential for AA to lead to UTUC [10]. As a result, the high prevalence of DNA adducts and mutations found in the upper urinary tract of patients exposed to AA may explain the reason why the chromosomal aberrations in UTUC specimens were more complex than those in UCB specimens [49]. Besides, UTUC cases were further found with more microsatellite instability and hypermethylation compared with UCB [50,51,52,53], while their frequencies of certain genomic alterations, such as alternations in the *TP53* and *CDKN2A* genes, also diverge [54]. These differences between UTUC and UCB not only highlight their different etiologies, but may also influence their therapeutic strategies.

## 3. The Distinct Clinical Modality of UTUC

Besides their distinct etiologies and the resultant difference in mutations, UTUC may further differ from UCB in terms of clinical modality. When it comes to clinical diagnosis of UTUC and UCB, their diagnostic approaches have been different. Unlike UCB, the technical challenges of obtaining sufficient tissue through ureteroscopic staging for UTUC increase the difficulty in making an accurate diagnosis of stage T2 (muscle-invasive) or T3 (peripelvic fat, periureteral fat, or renal parenchymal invasion) disease regarding its depth of infiltration [55]. Therefore, whether to perform radical surgery or not is generally according to a combined risk stratification based on findings of computed tomography urography, ureteroscopic biopsy, cytology, as well as the presence of hydronephrosis and previous radical cystectomy history for bladder cancer [56].

These discrepancies between UTUC and UCB underline the rationale of developing distinct therapeutic approaches respectively. In general, the notion of local treatment with kidney function preservation is shared by low-risk UTUC and early-stage UCB [57,58]. However, for invasive high-risk UTUC, radical nephroureterectomy (RNU) with bladder cuff excision with or without template lymphadenectomy was considered as the standard of care [56], which is different from radical cystectomy, urinary diversion, and lymph node dissection for advanced-stage UCB [59]. This divergence in preserving kidney or not in advanced diseases further underpins the different concerns about their following adjuvant therapies.

When it comes to adjuvant chemotherapy for urothelial carcinoma, the evidence for cisplatin-based combination chemotherapy in UCB is fairly robust [60], while relatively limited, but supportive evidence approves of this benefit in UTUC [56,61]. However, it is important to point out that due to the compromised renal function following RNU of the invasive high-risk UTUC cases, they are more likely to be unsuitable for cisplatin-based therapies, which have been well known for their renal toxicity [62]. For instance, it was reported that in 388 patients who underwent RNU, their postoperative mean estimated glomerular filtration rate was 24% lower than their preoperative one, and this further excluded about one-third of eligible candidates for postoperative chemotherapy [63].

Although cisplatin-based combination chemotherapy is also the first-line treatment for both metastatic UTUC and UCB [64,65], more than half of these patients were likely to have compromised performance status, impaired renal function, peripheral neuropathy, or heart failure, which resulted in cisplatin ineligibility [66,67]. Moreover, despite the attempt to search for alternative first-line chemotherapies, previous carboplatin-based treatments showed inferior outcomes compared with cisplatin-based regimens in patients that can tolerate cisplatin [68]. A randomized trial of the EORTC study 30986 comparing two carboplatin-based combination therapies also reported that treating cisplatin-ineligible patients with both of these regimens may still result in substantial toxic effects [69]. In this study, severe acute toxicity, including death, grade 4 thrombocytopenia with bleeding, grade 3 or 4 renal toxicity, neutropenic fever, or mucositis, was observed in 9.3% and 21.2% of patients among the two arms, respectively, with both of their median overall survival rates being less than a year. As a result, the clinical modality of invasive high-risk or metastatic UTUC called for novel therapeutic agents and therefore paved the way to find immune checkpoint inhibitors as one of the most promising alternatives.

## 4. Immune Checkpoint Inhibitors in Cancer Treatment

In general, cell surface receptors that modulate immune responses of immune cells are classified as immune checkpoints, such as programmed cell death protein 1 (known as PD1) and cytotoxic T lymphocyte protein 4 (CTLA4). These molecules negatively regulate the immune system, preventing excessive and prolonged immune activation that may otherwise lead to unwanted tissue damage or even autoimmunity [70]. When we look into PD1, it is encoded by PDCD1, is expressed on T cells and pro-B cells [71,72], and exerts its inhibitory function on T cell activity after attaching to its ligands. However, tumor cells have been known to take advantage of this inhibitory pathway by presenting ligands for PD1 (PD-L1 and PD-L2) [73,74], forming their immunosuppressive tumor microenvironment accompanied by other immunosuppressive strategies [75,76,77]. Therefore, it has been postulated that blocking inhibitory checkpoint molecules such as PD1 and its ligands, PD-L1 and PD-L2, could restore the suppressed anticancer immune response [78]. This therapeutic strategy has developed into various inhibitory antibodies targeting immune checkpoints, which is known as immune checkpoint inhibitors (ICIs).

Starting from the first PD-1 inhibitor, pembrolizumab, approved by the Food and Drug Administration (FDA) in 2014, ICIs have gradually been implemented in the therapies against nine different cancer types [79]. For instance, in the KEYNOTE-002 study, they compared pembrolizumab and investigator-choice chemotherapy (ICC) for patients with unresectable stage III or stage IV melanoma that was ipilimumab- and/or BRAF inhibitor-refractory [80]. It revealed that there was no significant difference in overall survival between different regimens, but both arms of pembrolizumab demonstrated superior median progression-free survival when compared to chemotherapy. In addition, superior response rates and lower incidence of grade 3–4 treatment-related adverse events were observed in the pembrolizumab arms. The robust clinical improvement with tolerable side effects of ICIs led them to a variety of clinical trials thereafter.

To achieve optimal ICIs responses, it is believed that tissue-based assays for PD-L1 expression, which was known to be associated with increased tumor size, aggressiveness, and poor outcome in various tumors [81,82,83], could serve as one of the most important predictors for prognosis [84]. Other biomarkers that predict the outcome of ICI therapies include higher mutation load [85], gene expression profiling linked to interferon gamma signaling [86], as well as densities and subtypes of tumor-infiltrating cytotoxic T lymphocytes [87,88,89]. Since AA-related tumor has been known for its extraordinarily high overall mutation rate [90], UTUC plausibly serves as a potential indicator to optimize patient selection in ICI trails.

For the same purpose, past research has looked into the two major intrinsic subtypes distinguished in UC, namely luminal and basal subtypes [91]. Intriguingly, although basal subtype tumors were found with increased PD-L1 tumor cell expression and higher PD-L1 immune cell prevalence, their responses to the second-line PD-L1 inhibitor atezolizumab were much lower than those belonging to the luminal cluster II subtype [92]. This finding was further repeated by another first-line atezolizumab trial [93], suggesting that intrinsic molecular subtyping also plays a key role in predicting the response to ICIs. Interestingly, it has been known that genes representing luminal subtype are highly expressed in UTUC [94,95,96], which were also found to be associated with an elevated incidence of mutations in *TP53* signaling, a greater genomic instability, and in non-smokers [97]. However, whether these findings prospectively stand for better responses to ICIs or retrospectively relate to an exposure history to AA require being clarified.

To date, many cancers that have been effectively treated by ICIs share the prognostic factors introduced, and UC is no exception. It was reported that bladder tumor cells expressed relatively high levels (about 20–30%) of PD-L1 [98,99]. In addition, the abundance of DNA alterations in UC is known to be surpassed only by lung cancer and melanoma [100], and most UC lesions are also surrounded by tumor-infiltrating immune cells [101]. These rationales led to multiple clinical trials to treat UC with ICIs, including both second-line and first-line attempts.

## 5. ICIs as the Second-Line Therapy for UCB and UTUC

Although the overall objective response rate to first-line cisplatin-based therapy is acceptable, the vast majority of UC cases eventually progress. In light of the high mutation load with a number of these mutations linked to heightened immunogenicity [102], UC is a good candidate for ICI therapies. Looking back to the first FDA-approved immunotherapy, *Bacillus* Calmette–Guerin, it decreases the risk of UC recurrence through stimulating the immune response against tumor cells [103]. Currently, multiple ICIs have been proven to be beneficial in treating intractable UC cases, including pembrolizumab, atezolizumab, nivolumab, durvalumab, and avelumab [79]. However, there is no trial of ICI designed specifically for UTUC so far, with only a limited proportion of the trials for UC having reported their subgroup analysis divided by the primary cancer site. Herein, we first introduce two of these second-line ICI regimens for UC, which has also been tested as first-line attempts, and then summarize current knowledge of the response of UTUC to ICIs.

Pembrolizumab (also known as Keytruda) is an anti-PD-1 monoclonal antibody. Among its phase III KEYNOTE-045 trial [104,105], 542 patients whose UC had recurred after or progressed on a platinum-based therapy were enrolled regardless of the level of PD-L1 expression and were randomly assigned to pembrolizumab (200 mg every three weeks for two years) or ICC (between paclitaxel, docetaxel, or vinflunine). It revealed that the overall survival was significantly increased within the pembrolizumab arm compared with chemotherapy, with the median being 10.3 versus 7.4 months. The response rate was also higher in the pembrolizumab arm, accounting for 21.1% versus 11.0% in the chemotherapy arm. In addition, other significant advantages of pembrolizumab over chemotherapy were also observed in terms of the higher estimated rate of response with a duration of 12 months or longer, a longer time to deterioration in health-related quality of life analysis [106], as well as a lower rate of serious treatment-related adverse events. Despite the lack of a statistically-significant difference in progression-free survival, the estimated 18-month progression-free survival rates were 16.8% versus 3.5% in favor of pembrolizumab.

Another PD-L1 inhibitor discussed here, atezolizumab, was most famous for its IMvigor211 phase III trial [107], in which 931 patients with metastatic UC treated after platinum-based chemotherapy were randomly treated with either atezolizumab (1200 mg every three weeks) or ICC (between vinflunine, paclitaxel, or docetaxel). In this study, overall survival was also examined hierarchically in pre-specified populations according to PD-L1-expressional prevalence of tumor-infiltrating immune cells. Although they did not discover a significant improvement with atezolizumab in overall survival within the subgroup of 234 patients with PD-L1 expression no less than 5%, the median duration of response turned out to be longer with atezolizumab compared with chemotherapy (15.9 versus 8.3 months). Similar findings were noted in the intention-to-treat population (21.7 versus 7.4 months), with 13.9% of patients in the atezolizumab arm remaining on treatment compared with only 1.9% of those assigned to chemotherapy during a median follow-up of 17.3 months. As expected, a higher response rate was observed in patients with elevated PD-L1 expression within the atezolizumab arm, while this characteristic was also linked to better responses in the chemotherapy arm. Importantly, the incidence of grade 3–4 toxicity was lower with atezolizumab compared with chemotherapy (20% versus 43%), and so was the incidence of treatment discontinuation (7% versus 18%) among the intention-to-treat population. Therefore, the superior safety profile and its durable responses in line with the previous phase 2 data allowed atezolizumab to be utilized in this setting.

Among all the second-line trials reviewed, only three of them had reported their outcomes regarding the UTUC subgroup, accounting for about 15–22% of their UC cases. In the single-arm phase 2 atezolizumab trial, durable, but slightly inferior response was noted in the UTUC group compared to the UCB group [92]. The following phase 3 RCT of atezolizumab, IMvigor211, showed no significant improvement with atezolizumab in overall survival of the UTUC and UCB subgroups, which reflects their null result in the whole cohort [107]. However, whether other benefits of atezolizumab observed in this trial, such as longer median duration of response and higher percentages remaining on treatment, had differences between UTUC and UCB was not shown. The third trial, known as theKEYNOTE-045 trial, showed that both UTUC and UCB tend to benefit from pembrolizumab more [104]. However, in both RCTs discussed, their confidence intervals of the UTUC subgroups were relatively wide. If future trials could further expand the UTUC sample size and report more associations related to this subgroup, more precise comments could then be made regarding the differences in the responses to ICIs, both between and within each subgroup.

Besides the successful development of ICIs into promising second-line monotherapies for advanced or metastatic UC, there have also been efforts implementing ICI agents as a part of combination therapies, including combinations of ICIs and chemotherapy, as well as combinations of ICIs and other immunotherapeutic agents. For instance, a randomized phase I/II study called CheckMate 032 was dedicated to finding the difference in safety and efficacy between nivolumab monotherapy (3 mg/kg) and nivolumab plus ipilimumab (at two different combinations of dosages) for four cycles followed by nivolumab monotherapy in advanced or metastatic solid tumors [108]. Within this cohort, the results concerning 208 patients with advanced or metastatic UC previously treated with a platinum-based therapy were reported, demonstrating that one of the combination treatment courses was not only associated with significantly better antitumor activity, but also well tolerated. Therefore, an extended phase III study, known as CheckMate 901, has been actively under investigation.

There are also ongoing clinical trials implementing atezolizumab or pembrolizumab as second-line therapies for BCG-unresponsive patients with non-muscle invasive bladder cancers [109,110], with encouraging news for pembrolizumab (of 200 mg every three weeks for 24 months), resulting in a three-month complete response rate of 38.8% [111]. Provided these flourishing prospects to treat UC with second-line ICI agents, however, it is critical to point out that most, if not all, of these investigations performed their studies focusing on UC, other than UTUC or AA-associated UC. Based on the different pathogenic mechanisms identified that may influence the response to ICIs, we encourage future research to do subgroup analysis based on UTUC, as well as an exposure history of AA.

## 6. First-Line ICIs for UCB and UTUC

As discussed in the previous sections, about half of the UC patients were ineligible for cisplatin-based therapy. Provided with the rationale to test out ICIs as the first-line alternative therapy for cisplatin-ineligible patients, clinical trials of pembrolizumab and atezolizumab have been carried out with an eye on the subgroup analysis divided by primary tumor sites.

Starting from 2015, the phase II KEYNOTE-052 study of pembrolizumab (200 mg every three weeks for up to two years) enrolled 370 patients with advanced UC who were also not eligible for a platinum-based treatment [112]. Among these patients, 42% of them were performance status 2, while 50% of them were included due to renal impairment. At a median follow-up of 9.5 months, there were 7% complete responses and 22% partial responses documented, and the median duration of response had not been reached. In addition, although responses were seen in all of the categories of PD-L1 expression, the highest response was found within patients with a combined positive score no less than 10%. When it comes to the subgroup analysis regarding the primary tumor site, slightly better response rates were noted in UCB, which was 28% compared to 22% in UTUC. In order to further evaluate the comparative efficacy, as well as safety for first-line use of pembrolizumab in this scenario, a phase 3 KEYNOTE-361 trial has been under investigation.

On the other hand, the study project IMvigor210 initiated its enrollment in 2014, which is a multicenter, single-arm phase II study of atezolizumab use (a total dose 1200 mg provided every three weeks) as first-line therapy for advanced or metastatic UC in 119 cisplatin-ineligible patients [93]. Seventy percent of these patients had renal impairment, while 56% of them had at least one independent prognostic risk factor (known as Bajorin risk factors) utilized to predict survival in metastatic UC [113]. After a median treatment duration of 15 weeks with a period of 17.2-month median follow-up, the objective response rate was 23% in all patients, 39% in patients with UTUC, and 28% in patients with no less than 5% of PD-L1 expression on tumor-infiltrating immune cells, with complete responses observed in 11 patients (9%). The median overall survival was 15.9 months in all patients, but had not been reached in UTUC patients. These findings demonstrated a better response in UTUC patients, which was not related to baseline covariates or microsatellite instability. Importantly, this study also found that patients within the highest quartile of mutation load showed significantly longer survival compared to the other quartiles; whether this may be a hint for the better response in the UTUC subgroup requires further confirmation. Overall, the attempt to treat cisplatin-ineligible UC patients with first-line ICIs was shown to be fairly promising and safe in selected conditions, and the high mutation load of UC likely played a role in the good efficacy. Therefore, atezolizumab was approved by the U.S. FDA in April 2017 to become the initial therapy for patients who are not candidates for platinum-based chemotherapy.

If we compare two first-line ICI trials, a clear contrast between the responses of the UTUC subgroup is noted. Since the ICI-regimens shared the same therapeutic mechanism, this variation could have originated from the intrinsic differences between the UTUC cases. Whether the genomic differences or the exposure to AA play a role in differentiating their responses to ICIs remains to be elucidated. Moreover, despite the promising results in these early phase clinical trials, it is important to point out that early reviews of their following clinical trials [114], which were KEYNOTE-361 and IMvigor-130, found that patients eligible for platinum-containing chemotherapy randomized into the ICI-monotherapy arms with PD-L1 low status had decreased survival compared to patients who received cisplatin- or carboplatin-based chemotherapy. As a result, future research needs to further identify the correct population that may gain further benefits from therapies of ICIs.

Recently, a single-arm phase II study examined the effects of gemcitabine and cisplatin plus ipilimumab, an anti-CTLA4 ICI, in 36 chemotherapy-naive patients with metastatic urothelial cancer [115]. They were treated with two cycles of cisplatin-gemcitabine and then four cycles of cisplatin-gemcitabine plus ipilimumab, resulting in an objective response rate of 69%, with 61% with one-year overall survival. Intriguingly, patients with deleterious somatic DNA damage response mutations had a higher response rate. Reminiscent of the distinct pathogenesis that links to DNA-adducts, impairments in DNA repair, and concordantly increased mutation load of a considerable part of UTUC cases (Table 1), it is plausible to test out whether AA-derived DNA adducts or the increased mutation load could be implemented as a prognostic biomarker prior to ICI therapy.

## 7. Directions for the Refinement of Patient Selection

In light of the distinct etiology and genetic mutations of the majority of UTUC that may potentiate a better response to ICIs, it is plausible to suggest future ICI trials to not only focus more on the upper tract-subgroup, but also take AA-related exposure or biomarkers into serious consideration as their potential prognostic factors, especially in AA-endemic areas. Since there has not been any trial of ICI for UTUC that implemented AA-related factors, we herein discuss known factors that are linked to AA.

Although utilizing retrospective questionnaires to collect history related to AA-exposure is one of the most convenient ways, AA-exposure history is not necessarily the carcinogenic factor that led to current UC, let alone the imprecision of the method itself. So far, the presence of AA-derived DNA adducts and an aberrantly high percentage of A:T → T:A mutations are the most widely-accepted hallmarks to indicate AA-associated UTUC and UCB, as introduced in the previous sections. However, Hoang et al. pointed out that AA-derived DNA adducts may well be presented in UTUC cases believed to be caused by smoking rather than AA, whereas AA’s mutational signatures such as the concurrence of mutation load of ≥40 single-base substitutions and >35% A:T → T:A transversions remain a valid biomarker to identify AA as the causative carcinogen in UTUC [116].

Thanks to recent progress in genetic sequencing, distinct genetic mutations of AA-associated UTUC have been reported. Besides *TP53*, both *KDM6A* and *CREBBP* genes were frequently mutated in AA-associated UTUC cases across studies [90,116]. However, in comparison to genetic mutations discovered in UTUC cases within the non-endemic areas, these genetic mutations related to AA are not exclusive. For instance, Nassar et al. performed targeted exome sequencings covering 237 genes in 472 UC specimens across grades and primary tumor site [117]. Although high-grade UTUC was the least frequent subgroup to have a *KDM6A* mutation, the frequency was still around 20%; whereas the frequency of mutated *CREBBP* accounted for 16% of all the UC cases. On the other hand, if we further attempt to compare intrinsic subtypes of UCB with these mutations in AA-associated UTUC, Robertson et al. found a strong association between mutations of *KDM6A* and *FGFR3* in UCB [118]. Although the luminal-papillary subtype of UCB is characterized by *FGFR3* mutations and it was reported that the majority of UTUC in the non-endemic area also resembled this subtype of UCB [119], *FGFR3* mutations were noted in only 0–8% of AA-associated UC [10,90,116]. Recalling the distinct etiology and biology of UTUC, careful interpretations are needed when drawing an analogy between intrinsic types of UCB to corresponding mutations in UTUC, and further genetic and epigenetic examinations focusing on UTUC and AA-associated UC are warranted.

To sum up, we encourage future ICI trials to implement AA mutational signals for subgroup analysis in light of the high mutagenic potency of AA, especially in AA-endemic areas. Although mutations in certain genes are more common in AA-associated UTUC, current knowledge shows that it is insufficient for them to stand for this etiology specifically, and therefore, they may not serve as good biomarkers to select AA-associated cancers.

## 8. Conclusions

Exposure to AA contributes to a considerable proportion of UTUC and UCB, through forming DNA-adducts, downregulating DNA repair genes, and increasing the mutation load. Based on the rationale of being effective against tumors with high mutation load, ICIs have generally proven themselves to be safe and at least non-inferior compared to traditional chemotherapy for advanced or metastatic UC. Multiple successful clinical trials of ICIs have recently identified the associations of better prognosis with certain common traits shared by AA-exposed patients. However, there has not been direct evidence from any ICI trail focusing on UTUC as a disease entity or implementing AA-related biomarkers so far. In light of the need to refine patient selection through exploring prognostic predictors, studies that expand our knowledge to the intrinsic subtyping for UTUC, as well as ICI trials for UTUC that perform subgroup analysis based on these findings are warranted.

## Figures and Tables

**Table 1:** Comparisons between UCB and UTUC in terms of genetic mutation and currently-known clinical responses to ICIs.

Features	UCB	UTUC
AA-derived DNA adduct	Uncommon	Common
A:T → T:A mutation of the *TP53* gene	Uncommon	Common
Mutation load	High among various malignancies	Even higher in AA-associated UTUC ^1^
Objective response rate to first-line ICIs ^2^	17–28%	22–39%

^1^ It was reported that AA-associated UTUC harbors a mean of 753 mutations per tumor and an average mutation rate of 150 mutations per megabase [90,116]. ^2^ Different findings regarding the UTUC subgroup were noted between the KEYNOTE-052 and the IMvigor210 study [93,112], underlining the importance of further clinical research with refined patient subgrouping to clarify this.

## Abbreviations

UCB	urothelial carcinoma of the bladder
UTUC	upper tract urothelial carcinoma
AA	aristolochic acid
ESRD	end-stage renal disease
UC	urothelial carcinoma
RNU	radical nephron-ureterectomy
ICI	immune checkpoint inhibitor
PD1	programmed cell death protein 1
CTLA4	cytotoxic T-lymphocyte protein 4
FDA	Food and Drug Administration
ICC	investigator-choice chemotherapy
